# Revisiting the Conventional Extraction of Protein Isolates from Faba Beans: Recovering Lost Protein from Sustainable Side Streams

**DOI:** 10.3390/foods14111906

**Published:** 2025-05-28

**Authors:** Abraham Badjona, Robert Bradshaw, Caroline Millman, Martin Howarth, Bipro Dubey

**Affiliations:** 1 Advanced Food Innovation Centre, Sheffield Hallam University, Sheffield S1 1WB, UK; c.e.millman@shu.ac.uk (C.M.); prof.m.howarth@gmail.com (M.H.); 2 Bimolecular Research Centre, Sheffield Hallam University, Sheffield S1 1WB, UK; r.bradshaw@shu.ac.uk; 3 School of Engineering and Built Environment, College of Business, Technology and Engineering, Sheffield Hallam University, Sheffield S1 1WB, UK

**Keywords:** faba beans, protein recovery, FTIR, functional properties, extraction

## Abstract

As the global demand for sustainable protein sources grows, valorizing side streams in plant protein processing has become crucial. This study revisits the conventional alkaline–isoelectric extraction of faba bean protein isolates, introducing an enhanced mass balance-driven approach to recover underutilized protein fractions from typically discarded side streams. Through strategic pH manipulation and centrifugation, four distinct protein fractions were recovered with purities ranging from 34.6% to 89.6%, collectively recapturing a significant portion of the 16% protein loss in standard processing. SDS-PAGE and FTIR analyses confirmed the structural diversity among the recovered fractions, with albumin-rich and globulin-rich profiles exhibiting unique spectral and electrophoretic signatures. Functionally, fractions B and D exhibited superior water- and oil-holding capacities, indicating their potential utility in food formulations requiring enhanced moisture and lipid retention. In contrast, fraction C, characterized by low water-holding capacity and high solubility, may be better suited to applications prioritizing emulsification performance, such as in dairy or meat analogs. This study not only highlights the feasibility of reclaiming high-quality protein from industrial byproducts but also underscores the potential of these recovered proteins in diverse food and non-food sectors, including pharmaceuticals and cosmetics. These findings contribute to circular economy strategies by transforming waste into value-added ingredients with functional and commercial significance.

## 1. Introduction

The global population is projected to grow by 40% by 2050, reaching around 10 billion people. However, currently, about 1 billion individuals are facing food insecurity [1]. Ironically, approximately 1.3 billion tons of food are wasted annually, leading to a carbon footprint of 3.3 billion tons of CO_2_ equivalents and the loss of significant quantities of water [2,3]. With a growing population, global food processing systems must expand and adapt as natural resources become scarcer, unless effective mitigation strategies are implemented [4].

Sustainable and cost-effective food production is a crucial challenge in tackling this pressing issue. Both industrial and academic professionals bear significant responsibility in managing and developing innovative methodologies to address this emerging trend. The biological conversion, extraction, and purification of valuable compounds from food byproducts are crucial for achieving “zero waste” in food production systems [5]. Utilizing pulses and legumes as a protein source reduces the escalating greenhouse gas emissions associated with animal-based protein sources like meat, dairy-based products, and eggs [6]. Moreover, the requirements of water and energy input for the production of plant-based proteins are much lower in comparison with animal-based proteins [7]. In protein production and purification processes, circularity and sustainability are rarely prioritized [8,9].

Most research on the extraction and technological properties of plant proteins primarily focuses on protein-rich sources such as pea and soybean, with comparatively fewer studies on faba beans [10,11,12]. Storage proteins are typically classified based on the Osborne classification system into four categories: water-soluble albumins, salt-soluble globulins, alcohol-soluble prolamins, and acid- or alkali-soluble glutelins [13]. In commonly utilized plant protein sources, such as pulses, albumins constitute approximately 10–20% of the total storage proteins, whereas globulins account for 60–80% [14,15]. During the wet alkaline–isoelectric precipitation method, the milled plant material is dispersed in an alkaline medium (pH 8–13), which facilitates the solubilization of storage proteins, including both albumins and globulins. The process then involves the removal of insoluble components via centrifugation, followed by pH adjustment of the solubilized proteins to their isoelectric point, typically within the pH range of 4–5 [16]. Under these conditions, globulins precipitate, whereas albumins predominantly remain in the soluble fraction. Subsequent centrifugation results in a protein-rich insoluble pellet (globulins), while the supernatant contains albumins along with other solutes such as sugars, phenolic compounds, and minerals [17,18].

This extraction approach is widely applied in both academic and industrial settings and demonstrates a primary focus on the utilization of globulin fractions. However, this conventional method generates a substantial side stream containing albumins, which is largely discarded. Given the processing scale, valorizing these side streams is critical, as demonstrated in soy protein isolate production, where the generation of 1 ton of soy protein isolate results in approximately 20 tons of side streams (soy whey) containing 0.3% (*w*/*v*) albumin protein [19]. Based on the protein composition of pulses, it is estimated that for every kilogram of globulin extracted, approximately 0.1–0.4 kg of albumins is produced [17]. The limited utilization of albumins in conventional extractions is attributed to the diverse protein composition of pulses and the variability in isoelectric precipitation pH, making their recovery more complex.

While the application of purified ingredients is justified in specialized applications, such as infant formula and nutritional beverages, to ensure consistency and predictable processing behavior, these ingredients are frequently recombined with other purified components in many formulations. A sustainable approach may involve utilizing minimally processed ingredients that naturally contain diverse techno-functional biomolecules, including proteins, fiber, and lipids. This strategy presents a promising means to reduce the environmental footprint of food production [20], particularly when derived from food processing side streams, which are generated in substantial quantities. Most recent studies investigating different types of faba bean protein have primarily focused on protein concentrates (~60% protein) [21] or protein isolates (80–92.2% protein) [22,23]. The use of sustainable pulses in industrial processes to generate a variety of protein ingredients results in the significant generation of byproducts containing valuable compounds [24,25]. To achieve a more sustainable food production system, the valorization of processing side streams is crucial, requiring prior assessment of their potential applications in food systems. The recovery and application of such valuable compounds from protein extraction side streams could create novel ingredients with economic opportunities and applications spanning the food, pharmaceutical, and cosmetic industries. Additionally, repurposing waste into value-added products may generate new revenue streams, contributing to economic growth.

Thus, to establish a circular economy through the utilization of side-stream biomass, exploration of the production stages is needed to manage unavoidable food waste and evaluate its potential applications. This article explores the conventional extraction process of isolates from faba beans as well as recovered proteins from side streams following different process conditions. Mass balance was carried out to track protein levels at all stages. The selection and control of extraction conditions was evaluated based on their effectiveness in maximizing protein recovery while preserving structural integrity. Extraction efficiency can be evaluated by assessing protein purity and yield, alongside monitoring structural modifications occurring throughout the process.

## 2. Materials and Methods

### 2.1. Raw Materials and Chemicals

Faba beans were purchased from Whole Foods Earth (Kent, UK). NaOH, (≥99.9% pure), β-mercaptoethanol, phosphate-buffered saline (PBS), and HCI were purchased from Sigma-Aldrich (United Kingdom).

### 2.2. Alkaline–Isoelectric Precipitation of Faba Bean Protein Isolates

The preparation of faba bean isolates followed the standard protocol described by Sheikh et al. [26]; Shen et al. [27] with modifications. Whole faba bean grains were dehulled and subsequently milled into flour using a laboratory-scale cyclone mill (Retsch, Twister) equipped with a 0.5 mm sieve. The particle size distribution of the milled flour was analyzed in replicates using a Mastersizer 3000 laser scattering particle size analyzer (Malvern, UK) with a dry sampling system [28].

**Figure 1:**
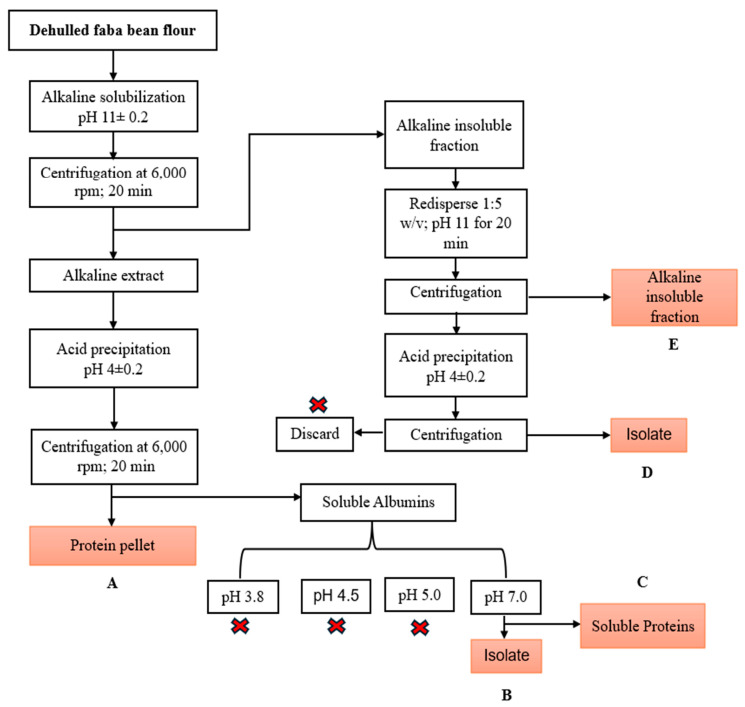
Flowchart for recovering protein isolates from faba bean flour.

Faba bean protein isolate (FBPI) was prepared as follows: 50 g of flour was dissolved in 500 mL of water (1:10 *w*/*v*). For alkaline solubilization, the suspension was stirred at 400 rpm using a magnetic stirrer for 20 min and adjusted to pH 11 using 1 M NaOH. The mixture was centrifuged at 6000 rpm for 20 min at 25 °C. After centrifugation, the supernatant was collected and adjusted to pH 4.0, using 1 M HCI while stirring for 30 min. Protein isolates were extracted by centrifugation at 6000 rpm for 20 min at 25 °C. The precipitated isolates were freeze-dried for 48 h, and the resulting protein was stored at −20 °C. Proteins in side streams from the starch fraction and isoelectric precipitation were further isolated using different pHs. The detailed process of the protein isolation process and conditions is shown in Figure 1. In general, dehulled faba bean flour underwent protein extraction through solubilization in an alkaline medium (pH 11 ± 0.2), which enhanced protein solubility. The alkaline-soluble fraction was separated from the insoluble material by centrifugation, and the resulting supernatant, containing solubilized proteins and other soluble components, was collected. Proteins were then precipitated by adjusting the pH to 4 ± 0.2, corresponding to the isoelectric point of globulin proteins. The precipitated proteins were isolated by centrifugation, forming a pellet designated as fraction A. The remaining supernatant, which contained soluble albumins, was further subjected to protein precipitation at different pH values (3.8, 4.5, 5.0, and 7.0) due to the complexity of the different protein subunits. Following centrifugation, protein pellets were obtained at each pH level, except at pH 7, where a protein pellet was successfully formed after centrifugation and designated as fraction B. The supernatant after the precipitation of fraction B contained soluble proteins and was labeled as fraction C. Additionally, the insoluble alkaline fraction from the initial centrifugation step was resuspended in water (1:5 *w*/*v*) and adjusted to pH 11 to recover any residual globulin proteins that may have remained associated with starch. This mixture was centrifuged, and the insoluble alkaline fraction was separated and labeled as fraction E. The resulting supernatant was further acid-precipitated at pH 4 ± 0.2 and centrifuged to isolate the precipitated proteins, which were designated as fraction D. The precipitated isolates were all freeze-dried, and the resulting protein was stored at −20 °C.

### 2.3. Protein Content and Extraction Yield

The protein content of the extracted protein isolates and side stream was determined using the Elementar Dumas system (Elemental, UK, Ltd., London, UK). A factor of 6.25 was used for the conversion of nitrogen content into protein content. The extraction yield was calculated by dividing the weight of the protein isolate obtained by the initial weight of the measured faba bean flour, as given in Equation (1):

(1) $$Extraction\;yield\;\left(\%\right)=\frac{mi}{ms}\;\times 100\%$$

(2) $$Protein\;yield\;(\%)=\frac{mi\times pi}{ms\times ps}\times 100\%$$

The mass of the extracted protein pellet and starting flour samples are denoted by *mi* and *ms*, respectively, while the protein content of the protein pellet and flour is represented by *pi* and *ps*, respectively.

The mass of the protein product and supernatant was determined after the centrifugation step following extraction. This was to identify which step proteins were lost and which recovered. The protein content of the supernatant and the pellet was measured. The protein of the faba bean flour is either extracted into the supernatant or stays in the plant cell and is lost in the pellet. If extraction is incomplete, residual proteins are found in the discarded supernatant or starch pellet.

As shown in Figure 1, the recovered proteins are represented by B, C, D, and E (loss to starch fraction) in all the extraction methods. Thus, the protein in the pellet recovered or lost during each step is shown in Equation (3):

(3) L or R=mi×pi

The lost (L) or recovered (R) protein in percentage relative to the amount of protein in the faba bean flour was calculated according to Equation (4):

(4) $$L\;or\;R\;(\%)=\frac{mi\times pi}{ms\times ps}\times 100\%$$

A mass balance was conducted from the protein content and protein recovery data for all the different fractions as follows:

m_f_P_f_ = m_A_P_A_+ m_B_P_B_ + m_C_P_C_ + m_D_P_D_ + m_E_P_E_

P_f_, P_A_, P_B_, P_C_, and P_D_ represent the protein content of the flour, fractions A, B, C, and D, respectively; m_f_, m_A_, m_B_, m_C_, and m_D_ represent the mass of the flour after freeze-drying, fractions A, B, C and D, respectively.

The sum of all the fractions should equate to 100%.

### 2.4. Qualitative Analysis of Proteins Using Electrophoresis (SDS-PAGE)

As per Laemmli [29], with some modifications, electrophoresis was carried out using SDS-PAGE in a reducing solution of β-mercaptoethanol. Briefly, 50 mg of protein powder was dissolved in 10 mL of PBS buffer (0.01 M, pH 7) and shaken at 200 rpm for 2 h at room temperature. Next, 10 μL of protein solution was dissolved and vortexed with 10 μL of loading buffer (reducing solution containing 10% 2-mercaptoethanol). The samples were heated for 4 min at 95 °C, cooled, and centrifuged at 13,300× *g* for 3 min. An aliquot was injected into the pocket of the Bio-Rad 4% acrylamide stacking gel, and 20% acrylamide was used to separate the Precast Gels. DC separation at a current of 25 mA was performed for one hour at a voltage of 200 V for 35 mins. An SDS-PAGE pre-stained ladder ranging from 260 to 8 kDa was used as a standard marker. The gel was rinsed with water and stained sequentially with Coomassie blue and imperial stain. The destained gel was scanned using gel analysis software (Nugenius, UK).

### 2.5. Protein Oil- and Water-Holding Capacity

The oil- and water-holding capacities were determined according to the method by Yang et al. [30] with modifications. Faba bean protein isolate (1.0 g) was dispersed in 40 mL of distilled water for the water-holding capacity (WHC) and rapeseed oil for the oil-holding capacity (OHC). The mixtures were vortexed for 1 min at maximum speed (2500 rpm) before standing for 6 h at room temperature (20–23 °C). Samples were then centrifuged at 3000× *g* for 30 min at 20 °C:

$$WHC/OHC=\frac{W0-W1}{W3}100\%$$

where W0 is the mass of the tube, the protein isolate, and absorbed water or oil; W1 is the mass of the tube and the protein isolate, while W3 is the mass of the faba bean protein.

### 2.6. Fourier-Transform Infrared Spectroscopy Analysis (FTIR)

A Fourier-transform infrared (FTIR) study was carried out using an attenuated total reflectance (ATR)-FTIR spectrophotometer (Spectrum 100 PerkinElmer, Murrieta, CA, USA). Prior to the experiment, the absorbance spectrum of the air was recorded and automatically removed from the sample spectra. Spectroscopic studies were carried out with freeze-dried faba bean protein isolates in the range of 4000 to 650 cm^−1^ at a resolution of 4 cm^−1^ with 16 scans.

### 2.7. Statistical Analysis

All statistical analyses were performed by Origin 2019 and Excel 2024 (version 2406). All the values were expressed as means ± standard deviation (SD). All the analyses were carried out in replicates (triplicates).

## 3. Results and Discussion

### 3.1. Extraction Yield

**Figure 2:**
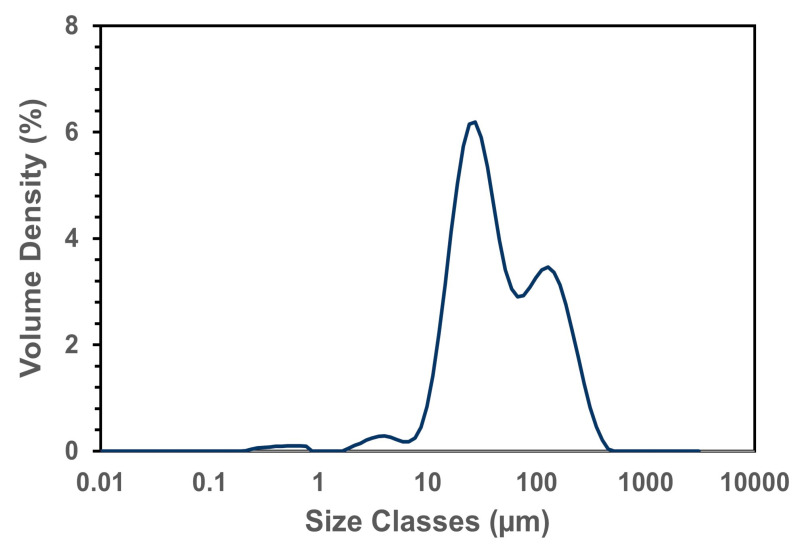
Shows the average particle size distribution of faba bean flour.

Extraction efficiency can be determined by tracking protein purity and yield, while structural changes were also observed during the extraction process. The first processing step in the fractionation process was grinding. This step is essential, producing fractured cells that liberate the starch granules and protein bodies [31,32]. Figure 2 and Table 1 show faba bean flour ground to a mesh cutoff size of 0.5 mm. The smaller classes may be associated with mainly protein bodies.

**Figure 3:**
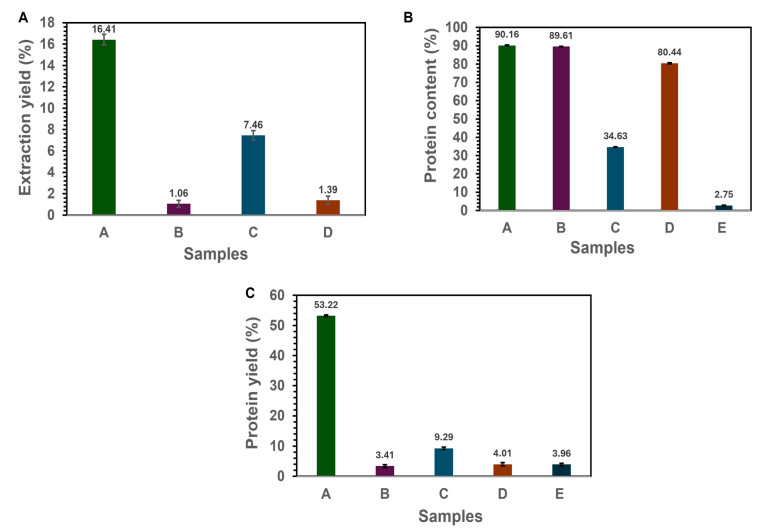
Extraction yield (**A**), protein content (**B**), and yield (**C**) during extraction; the yield of the four different side streams; and the resulting isolates (*n* = 3).

The effect of each purification step on the composition was studied in the milled faba bean protein extraction process. The first step was grinding the faba beans into flour. This flour was considered the initial starting point with a protein content of 27.29%. The first functional fraction was labeled as fraction A from the usual extraction process and resulted in an extraction yield, protein yield, and protein content of 16.41 ± 0.50%, 53.22 ± 0.30%, and 90.16 ± 0.25%, respectively. Fractions B and C were obtained from soluble albumins mostly discarded during protein isolation. Fraction B was obtained by simple precipitation at pH 7 of the soluble albumin solution (pH 3.8, 4.5, and 5.0 were investigated; however, no extract was obtained) and subsequent centrifugation to obtain protein pellets, while the remaining supernatant was labeled as fraction C (soluble proteins). Fraction B resulted in an extraction yield, protein yield, and protein content of 1.06 ± 0.32%, 3.41 ± 0.43%, and 89.60 ± 0.10%, respectively. Fraction C, after freeze-drying, showed an extraction yield, protein yield, and protein content of 7.45 ± 0.37%, 9.29 ± 0.40%, and 34.63 ± 0.12%, as shown in Figure 3. Fractions D and E were obtained from the alkaline insoluble residues that are usually discarded after alkaline solubilization and centrifugation. Alkaline insoluble pellets were redispersed in distilled water, and the pH was adjusted to 11. This was stirred and centrifuged to obtain a supernatant and alkaline insoluble fraction (E). The supernatant was adjusted to pH 4 to precipitate the residual proteins represented as fraction D. The supernatant was then discarded. The step-by-step extraction and recovery process is fully illustrated in Figure 1. Fraction D showed an extraction yield, protein yield, and protein content of 1.39 ± 0.37%, 4.01 ± 0.52%, and 80.44 ± 0.28%, respectively. Alkaline insoluble starch fraction (E) showed an extraction yield and protein content of 40.13 ± 0.43% and 2.75 ± 0.15%. The fat content of the faba bean flour used in the study was less than 2%, and it is assumed that the fractions contain a negligible amount of oil [33]. The higher extraction yield of fraction C (7.46%), with a low protein content compared to fraction B (lower extraction yield of 1.06% and protein content of 89.61%), indicates the presence of impurities, such as soluble sugars, minerals, phenols, and soluble fibers, after precipitation at pH 7. Additionally, at pH 7, only specific proteins were extracted into insoluble protein pellets, explaining the major difference in yield and protein content. The protein yield (% of recovered protein expressed over the initial protein content of the starting material) of the globulin fractions (A + D) was 57.23%, and for the albumin fractions (B + C), it was 12.7%. This is in line with globulin and albumin compositions in pulses. The protein purity of the faba bean isolate (A), labeled fractions B, C, and D, is consistent with that reported in the literature using a similar extraction process [22,34]. While some of the recovered fractions, particularly fraction C (protein content ~34.6%) and fraction E (~2.75%), exhibited minimal protein concentrations, these are not protein-pure isolates and are expected to contain other co-extracted or residual constituents. The remaining mass is primarily attributed to polysaccharides because of the starch-rich nature of the source material and the processing steps involved (especially in the case of fraction E). A significant portion of these fractions likely consists of residual starch and soluble polysaccharides not completely separated during centrifugation or precipitation. This is supported by FTIR data showing elevated absorption in the 1200–950 cm^−1^ region, which corresponds to carbohydrate vibrations. Additionally, although the overall fat content of the faba beans used was low (<2%), small quantities of co-extracted lipids or lipophilic compounds may persist in the matrix.

Mass balance for the different steps of protein extraction:

m_f_P_f_ = m_A_P_A_ + m_B_P_B_ + m_C_P_C_ + m_D_P_D_ + m_E_P_E_ + L_p_ (50 × 0.2779) = (8.2023 × 0.9016) + (0.52825 × 0.896) + (3.72695 × 0.3463) + (0.6932 × 0.8044) + (20.0626 × 0.0275) + L_p_ 13. 895 = 10.2681 + Lp Lp = 3.63 g

**Figure 4:**
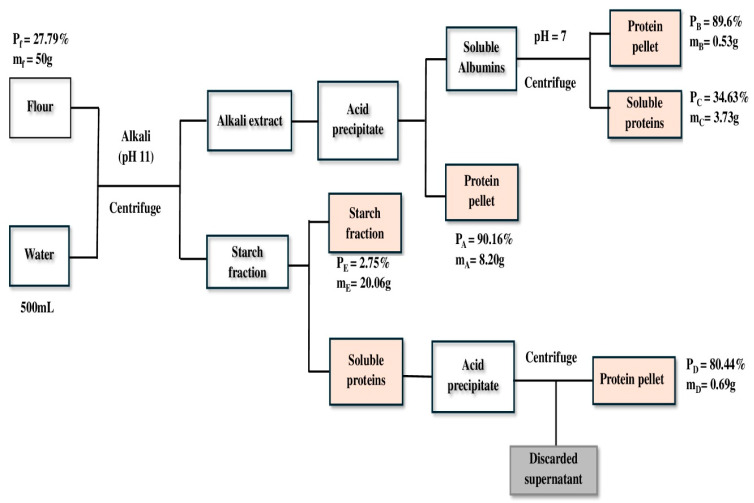
Schematic representation of the faba bean protein isolation process showing mass balance and protein balance. P_f_, P_A_, P_B_, P_C_, and P_D_ represent the protein content of flour, fractions A, B, C, and D, respectively; m_f_, m_A_, m_B_, m_C_, and m_D_ represent the mass after freeze-drying for flour, fractions A, B, C, and D, respectively.

Thus, the general loss to the extraction process (Lp) was 3.63 g, representing 26%, which may be attributable to the discarded supernatant. The general mass and protein balance is shown in Figure 4.

### 3.2. Qualitative Analysis of Proteins Using Electrophoresis (SDS-PAGE)

**Figure 5:**
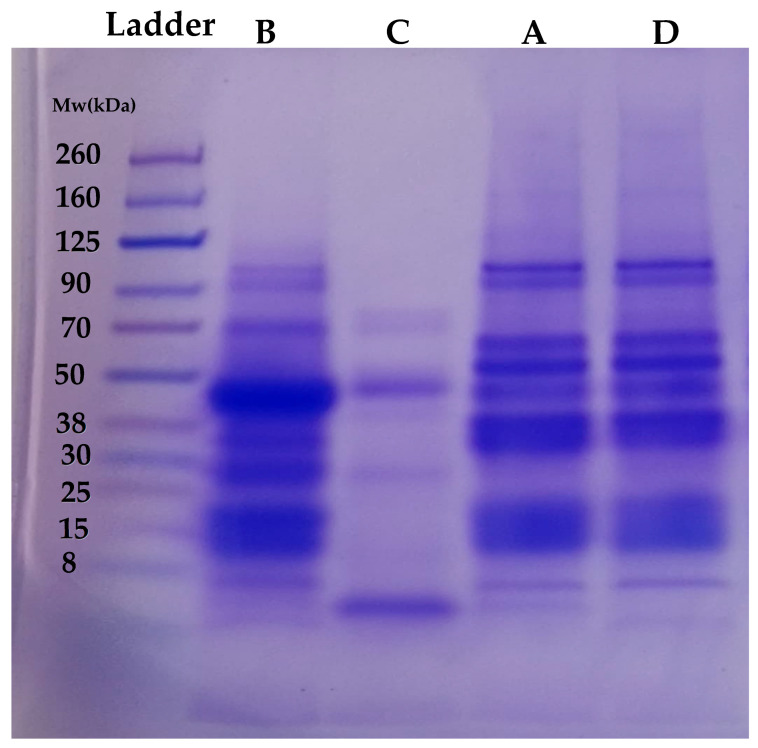
SDS-PAGE gel visualization of the different protein fractions under reducing conditions.

The primary structure of FBPI and the side stream recovered were analyzed by the patterns from reducing SDS-PAGE (Figure 5). Bands of the different proteins generated (A–D) showed different protein profiles. A similar SDS-PAGE profile was observed between A and D, which is not surprising since D represents the remaining globulin fraction extracted from the discarded starch fraction. Protein bands ranging from 260 to 8 kDa were observed in A and D. Both A and D were extracted using an Ip of 4.0 and thus were mostly composed of globulin fractions, hence the similarity in SDS bands. Major bands were observed at 15, 30–38, 50, 60–70, 90, and ~125 kDa for samples A and D. Smearing bands were also observed from 260 to 125 kDa. Samples B and C represent albumin-soluble fractions that are usually discarded after protein precipitation. In Figure 5, major differences in protein profiles can be observed between fractions B and C despite being from the same albumin solutions. Sample B showed a protein band of 8~90 kDa, while sample C showed a major band at only ~8 and 90 kDa. Additionally, minor bands were observed between 70–80 kDa and 30 kDa. Sample B contained a high protein content compared to sample C, which may indicate the presence of major protein bands. At ~50 kDa, both fractions B and C showed a band, but that of fraction B was more pronounced than that of fraction C. Similar studies on pulses, such as chickpea albumin fractions, have shown varying band intensities of 15–50 kDa [35]. Additionally, the SDS-PAGE profiles of soluble fractions of faba bean and pea proteins showed bands of 28–130 kDa. An observed band at ~55 kDa was attributed to vicilin subunits [36]. Mung bean albumin fractions have also been reported to have bands ranging from 14 to 50 kDa [17]. Comparing all the fractions, the globulin fractions (A and D) and the albumin fractions (B and C) showed differences in their protein profiles; however, albumin fraction B showed some similarity in bands to those of the globulin fractions. Interestingly, there was a band observed between 25 and 30 kDa in fraction B that was not found in any other fraction. Under the reducing conditions of SDS-PAGE, vicilin was found in its dissociated subunits of ~33–35 kDa (α and β) and 47–50 kDa (α, β, and γ), while legumin was observed in its acidic (α: 40 kDa) and basic (β: 20 kDa) subunits [37]. The observed band of ~70 kDa was presumed to correspond to convicilin [38]. Bands around 14 kDa have been reported to be a mixture of albumins and prolamins [39]. Overall, the globulin fractions (A and D) showed a different profile from those of the albumin fractions (B and C).

Fractions A and D displayed similar banding profiles, with major bands at 15, 30–38, 50, 60–70, 90, and ~125 kDa, consistent with globulin-type proteins. This similarity is attributed to their shared isoelectric precipitation at pH 4.0 and suggests the retention of structural integrity post-extraction. These fractions likely maintain the globular protein conformations essential for gelation, foaming, and water/oil retention functionalities. In the case of fraction B, derived from albumin-rich supernatant, it showed a wider range of bands (8–90 kDa) and the unique presence of a 25–30 kDa band not found in other fractions. This implies a broader protein population and the possible presence of low molecular weight peptides, which can influence solubility and digestibility and may support bioactive functionality. However, fraction C showed only dominant bands at ~8 and 90 kDa, suggesting a lower diversity of protein species and possibly a higher concentration of soluble albumins. Its limited protein complexity and higher solubility correlate with its low water-holding capacity, making it less suited for textural applications but more promising for emulsification, particularly in liquid or semi-solid food matrices.

### 3.3. Functional Properties

#### 3.3.1. Water- and Oil-Holding Capacity

**Figure 6:**
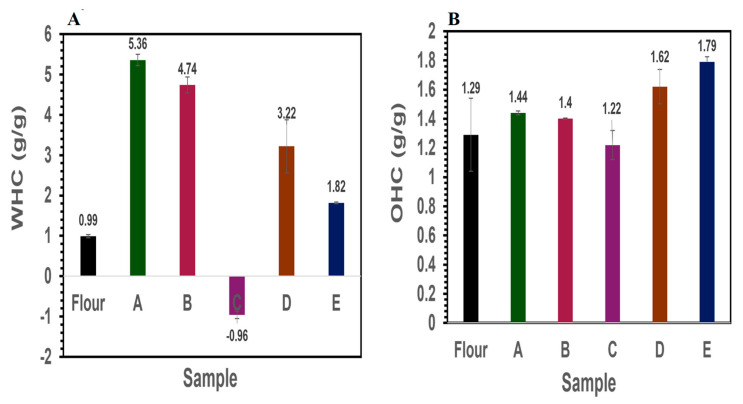
Water and oil- holding capacity of different protein fractions from faba bean flour.

Water-holding capacity (WHC) is defined as the quantity of water that can be absorbed per gram of protein. This is the affinity of water to bind to proteins via electrostatic interactions and is related to protein structure and the hydrophilic groups readily present to interact with water, while oil-holding capacity (OHC) represents the quantity of oil that can bind 1 g of protein [40]. Both WHC and OHC are affected by several factors, such as surface hydrophobicity, protein composition, particle size, and processing conditions [22]. Understanding the water- and oil-holding capacities of ingredients is particularly important in developing novel food products, such as plant-based meat analogs, eggs, and yogurt alternatives. The ability to retain these fluids plays a crucial role in developing the desired juiciness while preventing liquid separation, which could negatively impact the visual and sensory appeal [41,42]. The OHC of protein ingredients is of great interest for food applications, as it is reflected in the emulsifying capacity, which is relevant for products such as mayonnaise. From Figure 6A, major differences in WHC were observed among all of the different fractions. Faba bean flour showed low WHC compared to the protein samples, except for C. The higher water absorption of samples A and B as well as sample D could be attributed to the high protein content, which enables the absorption of protein to form a structured network. However, sample C showed a negative effect on WHC, which could be attributed to its high protein solubility at neutral pH. This observed negative water-holding capacity reflects its high solubility rather than a true inability to hold water since this fraction remains soluble and added water would dissolve it rather than form a structured network compared to globulins. This high solubility may have advantages for certain food applications such as emulsions and beverages. The observed high solubility and lower water-holding capacity have been documented in the literature [43,44]. Comparable OHC results were evident, as shown in Figure 6B. The highest OHC was observed in starch fraction E (1.8 ± 0.04 g/g) and protein fraction D (1.6± 0.12 g/g). Protein isolates A, B, and C showed OHC values of 1.44 ± 0.01, 1.40 ± 0.01, and 1.22 ± 0.10 g/g, respectively. Raw faba bean flour was observed to have an OHC of 1.29 ± 0.25 g/g. This indicates the possibility of incorporating these side streams usually regarded as waste into different food systems to achieve a circular economy.

The observed variations in water-holding capacity (WHC) and oil-holding capacity (OHC) among the faba bean protein fractions (A–E) can be attributed to multiple compositional and structural factors influenced by the extraction process; fractions A, B, and D, which exhibited higher WHC and OHC, also contained higher protein purities (80–90%). A greater abundance of hydrophilic amino acids and intact protein structures facilitates stronger binding with water and oil molecules. In contrast, fraction C, with only ~35% protein content, showed poor WHC. The lower protein density, likely diluted with non-protein components such as carbohydrates, limits its capacity to bind water. Differences in the secondary structure confirmed by FTIR and SDS-PAGE might have significantly influenced their interaction with water and oil. For instance, globulin-rich fractions (A and D) are known to exhibit stronger water and oil absorption due to their compact tertiary structures and balanced hydrophilic–hydrophobic regions. Albumin-dominant fraction C, with its high solubility and compact molecular conformation, lacks sufficient surface area or structural features to retain water yet remains efficient in oil interaction during emulsification. The presence of carbohydrates and lipids in lower-purity fractions, such as C and E, may interfere with protein–water/oil interactions by physically obstructing or altering the protein matrix. The functional disparities among the fractions are a direct result of the extraction pH, degree of purification, structural integrity, and molecular interactions developed during processing. These findings emphasize the importance of tailoring the extraction parameters to optimize specific functional traits, depending on the intended end use (whether it is moisture retention, emulsification, or structural reinforcement in plant-based food systems).

#### 3.3.2. ATR-FTIR Spectroscopy

**Figure 7:**
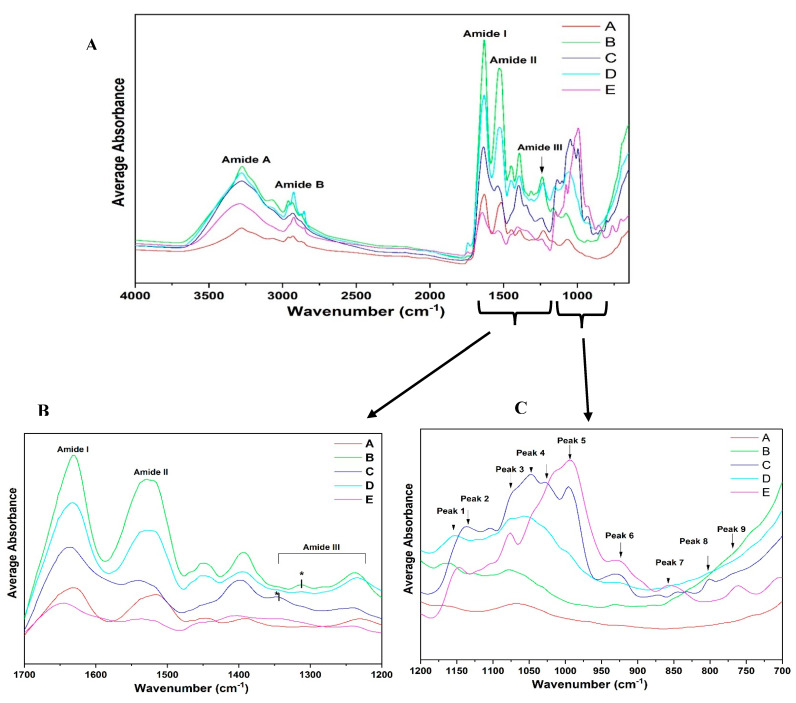
FTIR spectra of protein samples extracted from faba beans and their side streams: (**A**) original spectra (n = 3), (**B**) amide I-III region 1700–1200 cm^−1^, (**C**) fingerprint region 1200–700 cm^−1^. * Denotes peak difference in Amide III region.

Average spectra were acquired, which show the characteristic band distribution of different fractions (A–E) (Figure 7). It is important to note that the ATR-FTIR data presented in this study are exploratory in nature and intended primarily for qualitative compositional analysis. The spectral findings are not used in isolation to draw definitive conclusions about protein secondary or tertiary structures. Instead, they serve as complementary evidence to other structural analyses such as SDS-PAGE, helping to highlight compositional shifts across different protein fractions. In general, high absorbance was observed in the region of 1500–1700 cm^−1^ (amide I and II regions) [45] and 2700–3500 cm^−1^ (lipid and carbohydrate regions) [46], moderate absorbance at 1200–950 cm^−1^ (carbohydrate band) [47], and relatively low average absorbance at 950–700 cm^−1^ and 1200–950 cm^−1^ (Figure 7A). All the spectra of fractions A–E showed major peaks for amides I, II, III, A, and B. Similar peaks were observed in the starch fraction (fraction E), though it had relatively low protein content compared to the other protein fractions. The modes most widely used in protein structural studies are amide I, amide II, and amide III (Figure 7B). Major differences among the fractions can be observed in the protein amide regions and fingerprint region of 1200–700 cm^−1^. Starch fraction E in Figure 7B shows a poor absorption peak in this region due to relatively low protein content. However, observing the fingerprint region of 1200–700 cm^−1^, starch fraction E shows relatively high absorption peaks in the region of 1200–950 cm^−1^. Similar high peaks are observed for fractions B–D, but the peak of fraction A is low due to its high protein content. The fingerprint region of 1200–700 cm^−1^ clearly shows similar peaks and intensity between fractions C and E. This may be due to the low protein content and, possibly, the presence of carbohydrates and lipids in these two fractions. Fractions A, B, and D have similar peaks in the fingerprint regions, mainly due to the high protein content and lower amount of residual compounds. When looking at the carbohydrate region for the different extraction fractions of 1150–1000 cm^−1^ (Figure 7C), fractions A and B show low levels of absorbance, but they are more distinct for fractions C, D, and E. This region may be useful for differentiating these fractions. Conformational differences in both the protein regions and the fingerprint regions could be attributed to changes in the micro-environment [48] of the extracted fractions and the different protein components.

### 3.4. Qualitative Analysis of ATR-FTIR Spectra: Amide I Region

For the structural characterization of these intermediate steps in the extraction process, ATR-FTIR spectroscopy was employed. Given the sensitivity of infrared absorption to protein conformation, the amide regions of the infrared spectra serve as a valuable tool for both qualitative and quantitative assessment of protein secondary structures. To fully exploit the potential of ATR-FTIR spectra, each amide region was individually analyzed for all the obtained fractions.

**Figure 8:**
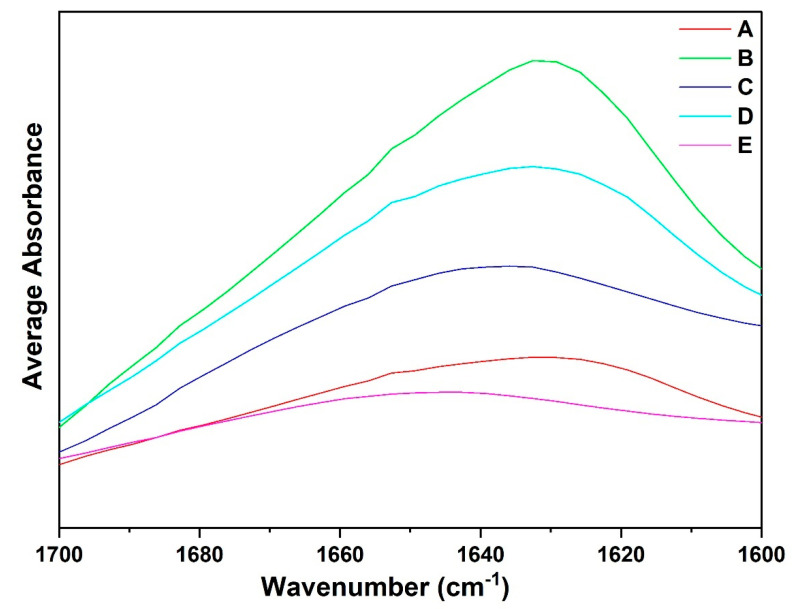
Amide I region of the faba bean isolate (A), and different side streams (B–E) (n = 3).

Analysis of all the spectra indicates notable absorption at higher wavenumbers, particularly in the amide I region (1600–1700 cm^−1^), which exhibits the highest sensitivity to conformational changes among all the amide regions. In contrast, variations in the adjacent amide II and III regions appear to be less influenced by secondary structure content. The amide I region primarily originates from C=O stretching vibrations and out-of-phase CN stretching vibrations within the polypeptide backbone [49,50]. Upon protein extraction, due to differences in protein content and secondary structure changes, spectral differences are very pronounced. Figure 8 shows the overlaid average spectra of protein fraction A and other recovered fractions B–E in the amide I region. The spectra of fractions A, B, C, and D display prominent peaks around 1640 and 1620 cm^−1^, while a minor peak was observed between 1660 and 1640 cm^−1^. Observing amide region I, each fraction spectra induced spectral changes. There is a shift in the peak absorption maxima of 1640–1620 cm^−1^. The average magnitude of the absorption of fraction A was lower than those of fractions B, C, and D. Generally, shifts in the amide I spectral region and differences in the magnitude of absorption could be attributed to the fact that fraction A may be mainly composed of globulin fractions, while fractions B, C, and D may be composed of albumin fractions.

### 3.5. Qualitative Analysis of FTIR Spectra: Amide II Region

**Figure 9:**
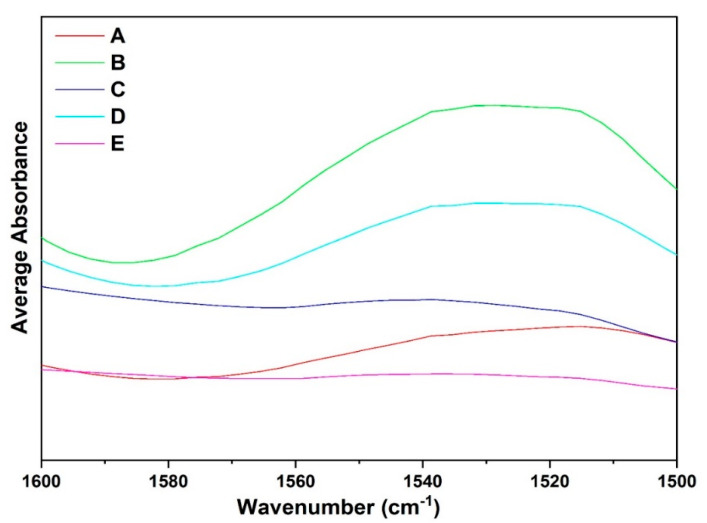
Amide II region of the faba bean isolate (A), and different side streams (B–E) (n = 3).

Regardless of the complete perturbations in the amide I region, the faba bean fraction spectra in the amide II conformational changes in the tertiary structure suggest that several amide NH groups are involved in strong hydrogen bonds and/or are buried within the hydrophobic protein core [35]. When comparing the different fractions, absorption maxima were observed around 1560–1500 cm^−1^ for all the samples except fractions C and E, which were mostly composed of starch and low levels of protein. Observing fractions A, B, and D, which had high protein content, shows major differences in the absorption maximum in this spectral region. Differences must be attributed to the high globulin content in fraction A compared to the other fractions, which may be mainly composed of albumin or other compounds. The results suggest that fractions A–D contained secondary structures with amide NH bond groups that are involved in stronger hydrogen bonds. Differences in the spectra of all the fractions reflect conformational variations in the tertiary structure among the samples, which show major spectral differences from 1500–1600 cm^−1^ due to differences in protein content and composition. The amide II region, which primarily results from NH in-plane bending and CN stretching vibrations, exhibits significantly lower sensitivity to specific secondary structures than the amide I region [2,36]. Due to its low sensitivity to secondary structure variations, the amide II region is well-suited as an internal reference for comparing amide I band intensities across different samples [37,38]. The spectral overlay for the amide II region, from 1600 to 1500 cm^−1^, has been provided for the different fractions extracted (A–E) (Figure 9). 

### 3.6. Qualitative Analysis of ATR-FTIR Spectra: Amide III and A–B Region

The amide III region is generally considered a less sensitive region within protein infrared spectra. Its bands primarily originate from NH bending and CN stretching vibrations, which exhibit some degree of conformation dependence [49]. Structural modifications influenced by variations in the extraction conditions, protein content, and chemical composition also lead to notable changes in this region.

**Figure 10:**
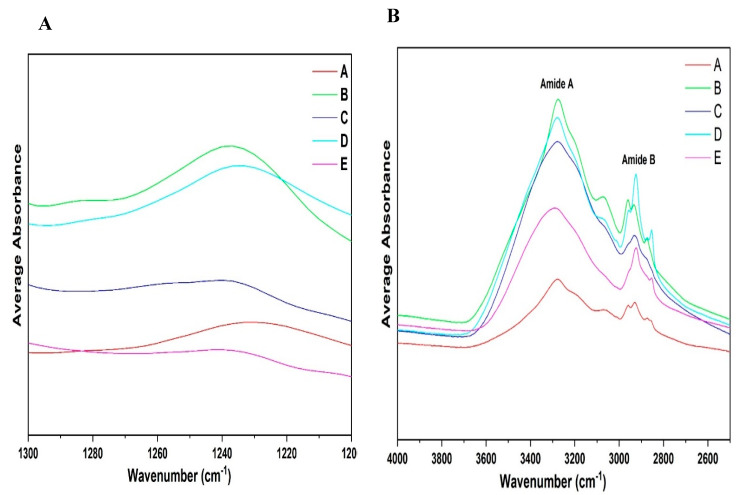
Amide III region of the different extracted fractions. (**A**) amide A and (**B**) amide B regions (n = 3).

The spectra shown in Figure 10 were analyzed based on the amide III band position ranges, as extensively studied by Cai et al. [51]. The amide I region (1700–1600 cm^−1^) is widely used due to its strong signal; however, it has limitations, including water interference, an unstructured spectral contour, and overlapping bands from various secondary structures. In contrast, the amide III region (1300–1200 cm^−1^), though weaker in intensity, is free from these limitations [51]. In recent years, several researchers have used the amide III region to determine protein structures [48,52]. Observing Figure 10A, the amide III spectra can differentiate between all the different fractions. A major peak was found in the region of 1260 and 1225 cm^−1^; however, this was less pronounced in fractions C and E due to their comparatively low protein content compared to fractions A, B, and D. Additionally, fraction A showed a lower absorption rate maxima compared to B and D. Figure 10A,B shows the differences in the amide A and B regions of the different fractions obtained.

## 4. Conclusions

A sustainable food production system requires valorization of its side streams, which have potential for different applications in the food, pharmaceutical, and cosmetic industries. The large-scale production of protein ingredients generates a significant volume of byproducts. In this study, we explored the maximization of the traditional alkaline–isoelectric process to obtain valuable side streams that are usually discarded. The results show the possibility of recovering lost proteins with a reasonable amount of protein content using different process conditions. Additionally, ATR-FTIR was applied to monitor the discrete compositional changes of each fraction that was obtained. Structural differences were observed in the amide I, II, III, A, and B regions of the fractions. SDS-PAGE analysis revealed different protein profile bands for all the protein fractions. Differences in the functional properties, such as the water- and oil-holding capacity, were observed for the individual fractions, indicative of the usefulness of specific side streams for specific food applications.

## Figures and Tables

**Table 1:** Particle size of the faba bean flour used during protein fractionation.

Particle Size of Flour		
D_x_ [10]	4.68 ± 0.1	μm
D_x_ [50]	29.4 ± 0.54	μm
D_x_ [90]	243.4 ± 2.61	μm
Span: (D90 − D10)/D50	8.12	-

## Data Availability

The original contributions presented in the study are included in the article, further inquiries can be directed to the corresponding authors.
